# An economic evaluation of a primary care-based technology-enabled intervention for stroke secondary prevention and management in rural China: a study protocol

**DOI:** 10.3389/fneur.2023.1145562

**Published:** 2023-05-02

**Authors:** Enying Gong, Bolu Yang, Xingxing Chen, Yuhan Li, Zixiao Li, Janet Prvu Bettger, Brian Oldenburg, Dejin Dong, Lei Si, Lijing L. Yan

**Affiliations:** ^1^School of Population Medicine and Public Health, Chinese Academy of Medical Sciences and Peking Union Medical College, Beijing, China; ^2^Global Health Research Center, Duke Kunshan University, Kunshan, China; ^3^School of Public Health, Wuhan University, Wuhan, China; ^4^Beijing Tiantan Hospital, Capital Medical University, Beijing, China; ^5^College of Public Health, Temple University, Philadelphia, PA, United States; ^6^Academic and Research Collaborative in Health, La Trobe University, Melbourne, VIC, Australia; ^7^NHMRC CRE in Digital Technology to Transform Chronic Disease Outcomes, Baker Heart and Diabetes Institute, Melbourne, VIC, Australia; ^8^Xingtai Center for Disease Control and Prevention, Xingtai, Hebei, China; ^9^School of Health Sciences, Western Sydney University, Campbelltown, NSW, Australia; ^10^Translational Health Research Institute, Western Sydney University, Penrith, NSW, Australia; ^11^Ningbo Eye Hospital, Wenzhou Medical University, Ningbo, China

**Keywords:** stroke, economic evaluation, cost-utility analysis, cost-effectiveness analysis, secondary stroke prevention

## Abstract

**Introduction:**

Secondary prevention of stroke is a leading challenge globally and only a few strategies have been tested to be effective in supporting stroke survivors. The system-integrated and technology-enabled model of care (SINEMA) intervention, a primary care-based and technology-enabled model of care, has been proven effective in strengthening the secondary prevention of stroke in rural China. The aim of this protocol is to outline the methods for the cost-effectiveness evaluation of the SINEMA intervention to better understand its potential economic benefits.

**Methods:**

The economic evaluation will be a nested study based on the SINEMA trial; a cluster-randomized controlled trial implemented in 50 villages in rural China. The effectiveness of the intervention will be estimated using quality-adjusted life years for the cost-utility analysis and reduction in systolic blood pressure for the cost-effectiveness analysis. Health resource and service use and program costs will be identified, measured, and valued at the individual level based on medication use, hospital visits, and inpatients' records. The economic evaluation will be conducted from the perspective of the healthcare system.

**Conclusion:**

The economic evaluation will be used to establish the value of the SINEMA intervention in the Chinese rural setting, which has great potential to be adapted and implemented in other resource-limited settings.

## Introduction

Stroke is one of the rising public health challenges worldwide. In 2019, there were ~12 million incident cases of stroke, of which 32.8% were in China (1). Although the stroke incidence rate is increasing, the stroke mortality rate has been decreasing over the past few decades, resulting in a huge number of stroke survivors globally (2). As these stroke survivors need continuous health management and risk control, the spending on post-stroke care causes an economic burden (3). The financial burden of stroke in rural areas is extremely severe (4, 5). As in many undeveloped areas, primary care lacks the capacity to provide guideline-based essential care to stroke patients, and community-based management for secondary prevention of stroke is far from adequate (6). Therefore, it is necessary to emphasize the prevention of recurrent strokes in the rural setting.

The system-integrated and technology-enabled model of care (SINEMA) study was designed to empower both stroke survivors and primary healthcare providers for secondary stroke prevention by training and incorporating both provider-facing and patient-facing mHealth technologies. The effectiveness of the SINEMA model has been evaluated and proven through a two-arm cluster-randomized controlled trial conducted in 50 rural villages of Hebei province in northern China (7). During the 1-year intervention, a statistically significant greater reduction in systolic blood pressure (SBP) was observed in the intervention arm compared with the control arm. Improvement in a few secondary outcomes including a 35–55% relative reduction in stroke recurrence, hospitalization, disability, and death has also been reported, which indicates the great potential benefits of the SINEMA intervention on secondary stroke prevention (8).

Despite indicating the effectiveness of the intervention, cost-effectiveness is another important factor to be considered. Previous economic evaluation studies of mHealth-based stroke prevention were reported to be cost effective. For example, the TEXT-ME trial conducted in Australia, a text message-based intervention for patients with cardiovascular diseases, reported that the intervention could gain 1,143 more QALYs and save a direct medical cost of Aus$10.56 million over a lifetime horizon for a hypothetical cohort of 50,000 patients with cardiovascular diseases in Australia (9). However, previous economic evaluations were mainly conducted in developed countries, and the cost-effectiveness of an integrated mobile health intervention on secondary stroke prevention in a resource-constrained setting like rural China remains unclear. Therefore, the economic evaluation of the SINEMA intervention is necessary. This protocol describes the methods for the economic evaluation of the SINEMA program based in a rural Chinese setting.

## Aim and objectives

This protocol describes the methods for the economic evaluation of the SINEMA program, which is nested in the SINEMA trial (8). This study aims to provide an economic evaluation of the SINEMA program to identify, measure, and value key resource and outcome impacts from the SINEMA intervention model compared with usual care for stroke secondary prevention in rural China. A within-trial economic evaluation will be conducted to calculate the within-trial incremental cost-effectiveness ratio to determine the value of the SINEMA intervention model.

## Materials and methods

### Study design

The economic evaluation is a nested study based on the SINEMA trial, a cluster-randomized controlled trial implemented in 50 villages in rural China. A detailed description of the SINEMA program and intervention design can be found in previous publications (7, 10–12). The economic evaluation will involve a within-trial cost-effectiveness analysis and cost-utility analysis with a 12-month time horizon equal to the follow-up period of the trial. We will calculate the incremental cost-effectiveness ratio in terms of the incremental cost per 1 mmHg change in systolic blood pressure, which is the primary outcome of the trial. In addition, we will also conduct a cost-utility analysis to calculate the incremental cost per quality-adjusted life year (QALY). The SINEMA intervention is deemed as cost-effective if the incremental cost per QALY is no greater than the cost-effective threshold. Following previous research (13), we will adopt the conventional approach by considering the benchmark as 1.5 times of gross domestic product per capita.

### Participants and study settings

Study participants in the economic evaluation will be the same as those recruited in the SINEMA trial. Participants were eligible if they were adults (older than 18 years), had a history of stroke diagnosed at a county or higher level hospital, were in a clinically stable condition with at least basic communication ability, and were expected to be available for the 12-month follow-up. Individuals who were unable to get out of bed had severe life-threatening diseases or had an expected life span shorter than 6 months were excluded. All participants were recruited in 50 villages from five townships in a rural county in Hebei Province, China. The county is a provincial-level impoverished county lying on the “stroke belt,” with a stroke burden double the national average level (10). Participants were recruited between 23 June 2017 and 21 July 2017 and followed until 27 July 2018.

### Intervention and control

The SINEMA intervention involved provider-side components and patient-facing components and was supported by a digital health system. In brief, village doctors, as primary healthcare providers, received training based on the train-the-trainer to train model. They were also equipped with the SINEMA app, they conducted monthly follow-up visits to patients. Additionally, financial incentives were also provided to encourage their ongoing commitment to deliver quality healthcare services. Stroke patients received monthly follow-up visits delivered by village doctors at the village clinics or their own homes if they had difficulty visiting the clinics. During each visit, they were provided with suggestions about medication use and physical activities. Participants who had access to their own or shared cell phones received one voice message, at no cost to them, for delivering health education information regarding medication adherence and physical activities.

For villages allocated to the control arm, village doctors continued their standard practices, which included practicing general clinical care and performing the “Basic Public Health Services” (BPHS). BPHS was announced when a new healthcare reform plan started in China in 2009, aiming at assisting community health organizations in delivering a set package of basic health services across the country (14). Patients in the control villages received the usual care. In the context of rural China, the usual care involved patients seeking care in village clinics, township healthcare centers, or county hospitals, as necessary. People with hypertension and diabetes may also receive quarterly follow-up visits by village doctors as covered by the Basic Public Health Services (10).

### Identification, measurement, and valuation of effectiveness

The intervention effectiveness will be measured by comparing the systolic blood pressure reduction and the QALYs between the intervention and control arm over the 12-month follow-up period.

#### Measurement of systolic blood pressure as the primary outcome

Blood pressure (BP) was measured as the primary outcome in the SINEMA trial at baseline and 1-year later, following the sample measurement protocol and approach among all participants. Blood pressure was measured on the right upper arm with participants seated and after 5 min of rest, with an electronic BP monitor (Omron HEM-7052). Two measurements were taken, and the mean value was calculated. If the difference between the two systolic BP measures was larger than 10 mmHg, a third measurement was conducted, and the mean value of the last two readings was calculated.

#### Health state utility

Health state utility (HSU) estimations will be derived from self-reported health-related quality of life (HRQoL) which was measured using the Chinese version of the EQ-5D-5L, a broadly used generic multi-attribute health utility instrument (15) at baseline and 1-year follow-up. For assessing HRQoL, study data collectors who were staff from the Center of Disease Prevention and Control in the nearby county read out the questionnaire and items for participants and collected the data. After answering the questions for EQ-5D, participants were asked to point out the health score by fingers on a paper version of the EQ-Visual Analog Scale, and then, the data collectors entered the responded values into the online survey platform. An HSU was calculated for each respondent by using the Chinese version of population-based preference weights (16), which ranged from −0.391 to 1, with 1 representing the value of full health, 0 representing deaths, and −0.391 representing the worst state.

Stroke recurrence, hospitalization, disability, and all-cause mortality were measured by using questionnaires at one-year follow-up. Medical and deaths records were also extracted from four major hospitals in the region. These data provide information about the status and trajectory of stroke during the trial period.

### Identification, measurement, and valuation of resource use and costs

The aim of the economic evaluation is to inform decision-makers about the costs and cost-effectiveness of introducing the SINEMA intervention to stroke patients in rural regions. As such, the economic evaluation will mainly be performed from the health sector perspective, reflecting the cost and values of the healthcare system.

The resources used to support the SINEMA program include as follows: (1) the cost used to support SINEMA program delivery and (2) the health resources used to support the healthcare service delivery to stroke patients. Table 1 describes the detailed measurement and valuation of costs. The research costs, including the investigator's time and data collection, were not included in the analysis. The costs of designing the SINEMA intervention and the digital health system and other “one-off” costs were excluded, but the operation and maintenance costs of the digital health system were included in the analysis.

**Table 1:** Resource use information collected and measured for cost.

**Cost component**	**Measurement**	**Quantity**	**Valuation**	**potential collected resource**
**Intervention related cost**				
Part-time project manager	Time spent coordinating the project	Number of project managers	Cost of employment converted to the annual equivalent cost	Research team to record
Materials for handbooks and handouts	Inventory on printing materials	Number of materials delivered	Replacement value converted to annual equivalent cost	Research team to record
County physicians	Time spent in training village doctors and providing consultations	Number of training sessions organized	Cost of employment converted to the annual equivalent cost	Research team to record
Township manager	Time spent in coordinating and delivering consultations	Number of townships participated	Cost of employment converted to annual equivalent cost	Research team to record
Village doctors	Time spent in delivering SINEMA follow-up visits	Number of villages participated and fidelity in adhering to the intervention protocol	Cost of employment converted to annual equivalent cost	Research team to record
Voice messages	Inventory on sending voice message via third-party platform	Number of voice message delivered	Replacement value converted to annual equivalent cost	Research team to record
Maintaining the digital health system	Inventory on server cost and cost related to human resources in maintaining the program	Duration of time supporting the program	Replacement value converted to annual equivalent cost	Research team to record
Developing digital health system	Time spent in designing the program	Number of staff involved	Cost of employment converted to annual equivalent cost	Research team to record
**Medical costs (for both intervention and control arm)**				
Inpatient cost	Time, human resources, facilities used for inpatient care	Mean days of stay and frequency of hospitalization within trial period	Mean cost replacement value recorded in social medical scheme	Reimbursement system
Outpatient cost	Time spent in delivering care	Number of follow-up visits	Replacement value converted to annual equivalent cost	(Estimated)
Medication cost	Self-reported medication use	Number of medications used and adherence rate	Replacement value converted to annual equivalent cost based on local medication price list	Follow-up survey

#### Resources used to support program delivery

Program costs are captured based on a detailed inventory of all resources that are used to support the design and delivery of the SINEMA program. This consists of the administrative cost of headcounts of local project manager and printing materials, the cash support that compensates for the time and efforts of the village doctors, township physicians, and county physicians in delivering the SINEMA intervention over the trial period, and the resources used for maintaining the digital component of the SINEMA intervention (including daily voice messages to patients, the mobile application server, and labor cost related to system maintenance).

#### Resources used for healthcare services

Medical costs are measured mainly by estimating the direct medical cost with individual-level data, including both inpatient costs and outpatient costs for medications. Inpatient costs during the trial period among all participants were retrospectively collected from the urban and rural resident basic medical insurance system from four major key hospitals in the region. A list of medical conditions, including cardiovascular or cerebrovascular events, or other cardiometabolic-related health conditions, is generated by researchers. All relevant inpatient records that matched the conditions will be included in the analysis and total costs will be used in the analysis. Outpatient costs were estimated by the number of hospital visits and medication use. Medication costs will be valued based on the general essential medication list and the standard unit cost for each type of medication as the “zero markups” regulation requires no additional costs on medications in the primary care settings (14).

### Data analysis

The data analyses will be performed using STATA software (StataCorp. 2019. State Statistical Software: Release 16. College Station, TX: StataCorp. LLC). For the within-trial cost-effectiveness analysis, two incremental cost-effectiveness ratios (ICERs) will be calculated to evaluate the incremental cost per QALY and the incremental cost per 1 mmHg reduction in systolic blood pressure.

The ICER formula is given below:


$$\begin{array}{l} ICER=\frac{\;Cost_{SINEMA}-Cost_{Usual\;care}}{Effect_{SINEMA}\;-Effect_{Usual\;care}\;} \end{array}$$

Multivariable or multilevel models (with levels defined as villages considering the cluster design) will be employed to explore factors associated with health resource use, cost, and effectiveness. Generalized linear regression modeling of costs with gamma distributions and log linked for multivariable analyses that adjust for age, gender, stroke type, and length of stay will be performed.

Several sensitivity analyses will be considered to quantify the level of decision uncertainty. Deterministic sensitivity analyses will be performed on chosen variables to identify key determinants for the results, as presented in Table 2. We will generate cost-effectiveness scatterplots to explore the uncertainties around incremental costs and effectiveness. A cost-effectiveness acceptability curve (CEAC) will be created to explore the probabilities of the SINEMA intervention being cost-effective at a range of cost-effectiveness thresholds.

**Table 2:** Sensitive analysis of key indicators.

	**Base case**	**Range**
**Effectiveness**		
SBP outcomes	Mean value in change with fully adjustment model	95% confidence interval for non-adjusted and fully adjusted
QALY outcomes	Mean value in change with fully adjustment model	95% confidence interval for non-adjusted and fully adjusted
**Cost**		
Compensation for village doctors	Recorded headcount cost	Fidelity rate of follow-up visits ranging from lowest value to 100%
Missing information of inpatient care for those who were hospitalized in other healthcare facilities.	Median inpatient cost for same or similar diagnosis	25%, 75% percentile of inpatient cost for those who were hospitalized with same or similar diagnosis.
Missing information of outpatient cost which is not covered by health insurance.	Mean outpatient cost for same or similar diagnosis	25%, 75% percentile of outpatient cost for those who were hospitalized with same or similar diagnosis.
Missing information on medication cost of daily doses.	Mean cost for the conventional drugs	25%, 75% percentile of medication cost for those who took same or similar drugs.
Development cost of the digital health system	None	Development costs of the SINEMA model when the cost was shared by years of follow-up

## Discussion

This manuscript details the study protocol of the economic evaluation that aims to assess the cost-effectiveness of the SINEMA intervention among stroke patients in rural China. As one of a few studies that evaluate the economic value of community-based technology-enabled intervention for stroke prevention and management, this study employs a within-trial evaluation to analyze the incremental cost-effectiveness ratios. If proven cost-effective, the findings from this study will provide robust evidence to policymakers in low- and middle-income countries for adopting and scaling up similar interventions.

Economic evaluation is a crucial component for evaluating the impact of a community-based intervention for disease prevention. Despite the proven effectiveness of the SINEMA intervention, the economic value of the SINEMA intervention will further inform decision-makers on the allocation of scarce resources for stroke prevention and control. Although effective strategies for community-based interventions have been increasingly examined through trials (17–19), only a few studies seek to answer the economic value of the intervention. For instance, the COBRA-BPS trial aimed to assess the effectiveness of community-based interventions for improving blood pressure control in Bangladesh, Pakistan, and Sri Lanka, and the COBIN study targeted lifestyle intervention for blood pressure control in Nepal estimated the long-term economic value through budget impact and cost-effectiveness analysis from the health system perspective (20, 21). However, these assumptions may introduce certain biases which may overestimate the benefit of the intervention (22, 23). Different from these studies, our study performed a within-trial economic evaluation method with individual granularity by using first-hand data collected from the trial. In addition, existing reviews also called for research to consider the uniqueness of measuring the cost and benefit of digital health solutions compared with traditional human-delivered interventions (24). Thus, our study may shed light on the field by detailing our methodology for evaluating the cost-effectiveness of a community-based technology-enabled intervention.

Our study design has several unique features. First, the study measures a multifaceted intervention with digital health components. Digital health interventions have a high potential to deliver interventions to a large-scale population, thus, it holds the promise of improving chronic disease management. Second, compared to human-delivered intervention, the development cost for digital health intervention is relatively high; however, the operation and marginal cost for the implementation could be limited to none if it is scaled to a large population (25). In our protocol, we followed previous studies' methods to evaluate the operational cost of the SINEMA model in our design (11). We also include the development cost in our sensitivity analysis.

As for data collection, due to the constrained funding support, the SINEMA trial observed and measured the cost and effectiveness of the intervention over a 12-month period. Although such follow-up duration was reasonable to measure a high number of stroke cases, the data collected from the trial period hardly provided information about the long-term benefits and impact of the intervention. Instead, we will use data from other trials and cohort studies that were conducted in similar settings to estimate the long-term natural transition of stroke patients in rural China (26, 27). This approach enables us to estimate the long-term economic impact of the SINEMA intervention.

The main limitation of this design lies in the field of cost measurement. First, although we measured key direct health costs at the individual level, we are only able to quantify the inpatient costs and medication costs for participants from the health insurance data. Due to the limited data access, we cannot measure the cost of inpatient care if the patients seek healthcare services beyond the major four hospitals in the study regions, which is likely to happen as there is no referral system that restricts service-seeking behaviors in rural China (27). Therefore, we tend to underestimate the inpatient costs if there are relatively more inpatients in the control group. Second, the analysis may underestimate some of the labor costs. For instance, the village doctors may provide blood pressure assessments during clinic visits for free, and the payment scheme may not be set by the number of services provided at the clinic visits. These visits were not paid, thus were not included in the financial analysis.

## Conclusion

This article details the study protocol of the economic evaluation of the SINEMA intervention. If proven cost-effective, the SINEMA intervention has a high potential to be adapted and implemented in other settings to benefit more people with stroke.

## Ethics statement

The studies involving human participants were reviewed and approved by Duke University, Beijing Tiantan Hospital. The patients/participants provided their written informed consent to participate in this study.

## Author contributions

EG and BY drafted the manuscript. EG, JB, BO, LS, and LLY performed the study design. XC, BY, LLY, and YL contributed to the manuscript revision. LS supervised the study design and data analysis plan. All authors approved the final version for publication.
